# Complementary and integrative medicine - Resolving situations of reduced remuneration for additional work under the SwissDRG system

**DOI:** 10.1016/j.heliyon.2024.e34732

**Published:** 2024-07-25

**Authors:** Fabian Grass, Chantal Berna, Charles-André Vogel, Nicolas Demartines, Fabio Agri

**Affiliations:** a Department of Visceral Surgery, Lausanne University Hospital CHUV, University of Lausanne (UNIL), Lausanne, Switzerland; b Center for Integrative and Complementary Medicine, Division of Anesthesiology, Department of Interdisciplinary Centers, Lausanne University Hospital, Lausanne, Switzerland; c Department of Administration and Finance, Lausanne University Hospital, Lausanne, Switzerland; d General Direction, Lausanne University Hospital CHUV, University of Lausanne (UNIL), Lausanne, Switzerland

**Keywords:** Complementary and integrative medicine, Cost weight, Diagnosis related group (DRG), Financial incentives, reimbursement (fee), SwissDRG grouping algorithm

## Abstract

**Aim of the study:**

Complementary and integrative medicine (CIM) has been increasingly recognized as offering promising treatment adjunctions in various clinical settings, even amongst patients with serious, chronic, or recurrent illness. Today, only few tertiary care facilities in Switzerland offer dedicated CIM services for inpatients. The aim of the present study was to evaluate whether CIM services for complex medical conditions are adequately valued by the national inpatient SwissDRG reimbursement system.

**Methods:**

A simulation was performed by adding a specific code of the Swiss classification of interventions (CHOP) to the list of codes of each patient who received CIM therapies at the Lausanne University Hospital (CHUV) in 2021. This code is to be used when CIM services are provided. Hitherto, it was not entered due to a lack of specific documents justifying the resources used. The analysis focused on the impact of adding this CIM CHOP code on the Swiss Diagnosis Related Group (DRG) reimbursement.

**Results:**

In total, 275 patients received a CIM therapy in 2021. The addition of the CIM CHOP code 99.BC.12 (10–25 CIM sessions per stay) resulted in a simulated loss of income of CHF 766 630 for the hospital, while the net real result is already negative by more than CHF 6 million. The DRGs positively impacted by the addition of CIM CHOP code 99.BC.12 had a mean (SD) cost weight (CW) of 1.014 (0.620), while the DRGs negatively impacted had a mean (SD) CW of 3.97 (2.764) points.

**Conclusion:**

It is necessary to quickly react and improve the incentives contained in the grouping algorithm of the prospective payment system, whose effects can threaten the provision of adequate medical care to the patients despite suitable indications and potential for cost-savings.

## Highlights

• Evidence shows that patients with complex medical conditions benefit from CIM services.
• Innovative care protocols such as CIM pathways need a reactive reimbursement strategy.
• Ensuring fair reimbursement is key to secure sustainable benefits for patients.

## 1 Introduction

Prospective payment system (PPS) was developed at Yale University in the 1970's, at a time when US nonprofit hospitals exhibited cost deviations of almost 100 % [1,2]. The underlying theory postulated that medical care costs were relatively predictable and responsive to shifting incentives, especially those aimed at reducing costs [3]. Prospective payment systems are case-based reimbursements and require a grouping algorithm to assign a case to its diagnosis related group (DRG). On January 1st, 2012, Switzerland implemented its PPS (SwissDRG) for inpatients. The current SwissDRG grouping algorithm uses diagnosis codes of the German modified international classification of diseases (ICD-10-GM), procedure codes (CHOP; Swiss classification of interventions) as well as clearly specified patient-related data such as gender, age and admission type. Each DRG has a resource utilization score known as the cost weight (CW). The predetermined fee per case is determined by multiplying the DRG's CW by the hospital's own base rate (BR). The BR is a fixed amount that converts the CW (points) into Swiss francs (CHF) and is typically renegotiated annually by each hospital [4].

Cases with length of stay (LOS) between limits individually fixed for each DRG are called “inliers”. Inlier LOS is statistically defined as “normal” for the DRG considered [5]. On the other hand, “outliers” have an “abnormal“ LOS for the DRG considered and are subject to special surcharges or deductions. High outliers are cases with a LOS higher than the upper limit of the DRG (high trim point, HTP) and trigger a surcharge. The surcharge is a daily compensation in terms of reimbursement. On the other hand, low outliers have LOS shorter than the lower limit (low trim point, LTP) and trigger a deduction, assuming less resources were used compared to the inlier patients of the same DRG [6,7].

The Swiss PPS creates incentives for providers to manage hospitalized patients in a cost-efficient way and enables performance comparison [8]. Cost weights are computed nationally for each DRG using the average costs of inpatients within the same DRG. Data from a preceding billing period are utilized for this calculation [4]. Nevertheless, Mehra et al. identified the difficulties in achieving reliable CW estimates for rare DRGs, since only inliers costs are used for their calculation [9].

In the last two decades, complementary and integrative medicine (CIM) has been increasingly recognized as promising adjunctive treatment in various ambulatory settings [10,11]. Implemented within hospital care settings, CIM have demonstrated advantages for patients alongside potential cost-saving opportunities [[12], [13], [14], [15]]. Furthermore, international guidelines recommend specific complementary approaches in the acute setting, e.g., acupuncture for the management of post-operative nausea and vomiting [16]. However, today, dedicated complementary medicine therapies are only offered in few tertiary Swiss hospitals, hence trailing behind our American counterparts [[17], [18], [19]]. Thus, integrative and complementary medicine serves as an illustration of a novel service integrated into the Swiss healthcare system, gaining traction among clinics and hospitals, including lately academic centres [[17], [18], [19]]. Therefore, the patient population benefiting from CIM therapies has evolved. As a result, its medical coding and reimbursement are currently facing considerable challenges in achieving consistent deployment of CIM services nationwide [[19], [20], [21]].

The aim of the present study was to evaluate whether CIM services for complex medical conditions are adequately valued by the national inpatient SwissDRG system for hospital reimbursement. In light of the increasing evidence demonstrating their values, ensuring fair reimbursement is key to ascertain sustainable and equalitarian access for patients.

## 2 Methods

All patients discharged between January 1, and December 31, 2021, and having received CIM therapies during their stay by the Centre for Integrative and Complementary Medicine (CEMIC) at Lausanne University Hospital (CHUV) were identified from institutional administrative data. Patients are referred to CEMIC by their ward's clinical team, mostly for managing pain, other acute symptoms, or anxiety related to their illness. Five years after the CEMIC's inpatient offer was made available to all CHUV wards, only a minority of the 55 000 annual patients treated as inpatients at CHUV are addressed to CEMIC, with a focus on symptoms that are refractory to conventional treatment and highly complex patients. CIM specialists evaluate referred inpatients' eligibility for CIM services and provide individualized strategies according to their specific needs if indicated.

Due to the lack of a specific justification strategy, no complementary medicine CHOP code (99.BC) was assigned to these patients. In fact, the code may be used only if the type, number and duration of each therapy per stay are clearly stated in the patient record, and that was not the case in 2021. The hospital-own electronic medical coding application (Medstat) was used to retrieve codes related to inpatient stays of all included patients. A simulation was performed by adding for each included case a CIM CHOP code to the codes effectively applied. Cases were grouped into DRGs using the official SwissDRG grouping algorithm 2021 [22].

The CHOP edition 2021 contains definite codes for CIM associated to the quantity of treatment units or sessions: code 99.BC.11 (1–9 CIM sessions per stay); 99.BC.12 (10–25 CIM sessions per stay); 99.BC.13 (26–49 CIM sessions per stay); 99.BC.14 (≥50 CIM sessions per stay). One session corresponds to 30 min of therapy, which encompasses treatments from these five domains: anthroposophical and Chinese traditional medicine, homeopathy, phytotherapy, and neural therapy. Furthermore, therapies must be supplied by a multidisciplinary team with the necessary credentials. Of note, the SwissDRG grouper algorithm never takes into account the CHOP code 99.BC.11 to classify a case in a DRG. This implies that at least 10 sessions of CIM therapies per stay are necessary for the service to be considered by the SwissDRG grouper. All other CIM CHOP codes are used by the grouper, which considers them to assign to a given DRG. Swiss DRGs specific for CIM are DRG A96B (complex CIM treatment, without surgical procedure, 10–25 sessions) and DRG A96A (complex CIM treatment, without surgical procedure, ≥26 sessions).

Anonymized data variables were collected using the hospital's electronic coding tool (Medstat), encompassing diagnostic related groups (DRGs) according to the 2021 definitions of the German modified international classification of disease (ICD-10-GM) along with their respective CW, hospital reimbursement amount (revenue) in Swiss Francs (CHF), and associated stay costs. Costs of the stays were obtained using a microcosting approach [23]. Medical coding data from Medstat were uploaded to the SwissDRG grouper 2021 for calibration [22]. The CIM CHOP code 99.BC.11, 99.BC.12 and 99.BC.14 were then successively added, and all cases were again grouped by the SwissDRG grouper 2021. The CHOP code 99.BC.13 was not considered useful for further study due to its intermediate nature between codes 99.BC.12 and 99.BC.14. Any conclusion drawn from the other codes is sufficient to understand the underlying incentives produced by the DRG grouping algorithm. Moreover, even if the hospital's medical coding centre was unable to actually code a 99.BC CHOP code, it was very common for the CEMIC to reach a minimum of 10 sessions per stay amongst patients with serious, chronic, and recurrent illness. Of particular interest was therefore code 99.BC.12, which would have been the most coded CIM CHOP code provided supporting documents were available. Our comparison focused on the DRG in which the cases were classified before and after the addition of the CIM CHOP code, their respective CW, the corresponding revenue and costs in CHF and their LOS.

## 3 Results

Over the year 2021, the CEMIC offered its services to 275 inpatients, whose mean (SD) age was 54 (16). The mean (SD) CW was 8.13 (11.8). Total costs amounted to CHF 32 979 109 and total reimbursement to CHF 26 709 105. Overall, the actual net result in the study population was a loss of CHF 6.3 million with a mean (SD) loss per case of CHF 22 800 (52 776).

Simulation with CIM CHOP code 99.BC.11.

Adding a CIM CHOP code 99.BC.11 (1–9 CIM sessions) to the initial medical coding had no impact on the grouping algorithm, and all cases stayed in their initial DRG.

Simulation with CIM CHOP code 99.BC.12.

Adding the CIM CHOP code 99.BC.12 (10–25 CIM sessions) had an impact in some cases. For 186 of the 275 patients (68 %), the CW was not affected by the CIM CHOP code 99.BC.12. These cases stayed in their previous DRG. In the remaining 89 cases (32 %), the initial SwissDRG was affected by the addition of the CIM CHOP code 99.BC.12. In 42/89 (47 %) of these cases, the new CW was lower, while it was higher in the remaining 47/89 (53 %) cases.

The addition of the CIM CHOP code 99.BC.12 generated a loss of revenue of 71.984 points of CW or a total loss of CHF 766 630. The largest single positive effect of adding a CIM CHOP code 99.BC.12 was +0.426 point of CW (mean: +0.239; range: +0.007 to +0.426), while the largest single negative impact was −7.865 points of CW (mean: −1.815; range: −7.865 to −0.007). When factored into the SwissDRG grouper, the CIM CHOP code 99.BC.12 consistently altered the original DRG to DRG A96B (with an inlier CW of 1.027).

**Fig. 1:**
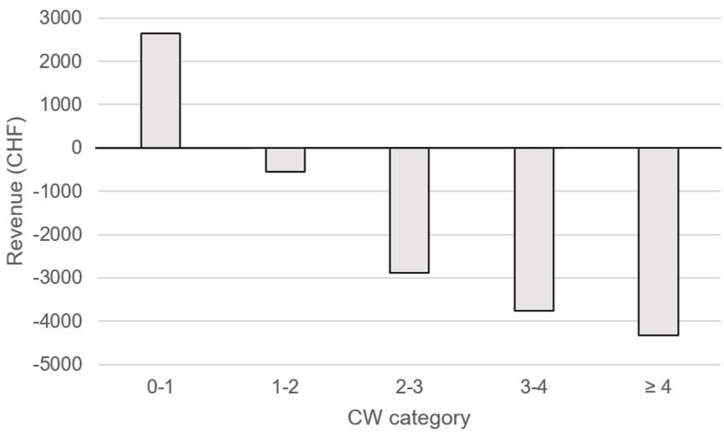
Impact of adding CIM CHOP code 99.BC.12 on cases revenue according to case complexity (i.e. Cost weight, CW).

The DRGs positively impacted by the CIM CHOP code 99.BC.12 had a mean (SD) CW of 1.014 (0.620). The DRGs negatively impacted had a mean (SD) CW of 3.97 (2.764) points. This effect is illustrated in Fig. 1, showing an increasing negative revenue gap with increasing CW categories after the addition of a 99.BC.12 CHOP code.

Among the 89 cases re-assigned to DRG A96B after adding CIM CHOP code 99.BC.12, 43/89 (48 %) were inlier cases and 46/89 (52 %) were outliers, among which 40 (87 %) were high outliers and 6 (13 %) low outliers. All 6 low outliers were also low outliers in their previous DRG. Fig. 2a, Fig. 2b, Fig. 2cA, 2B and 2C show examples of cases pushed into DRG A96B.

**Fig. 2a:**
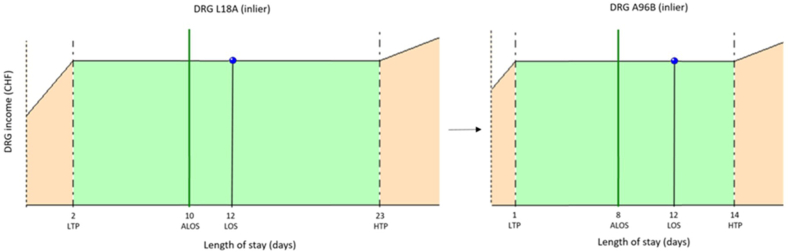
Examples of impact when adding CIM CHOP code 99.BC.12 From SwissDRG L18A inlier to SwissDRG A96B inlier. The initial DRG L18A (complex transurethral and percutaneous transrenal procedures and other retroperitoneal procedures without extracorporeal shock wave lithotripsy, with severe level of complication or comorbidity) with an inlier CW of 1.748 point (CHF 18 616) is pushed into DRG A96B when CHOP code 99.BC.12 is added to the list of codes, resulting in an inlier CW of 1.027 point (CHF 10 938).

**Fig. 2b:**
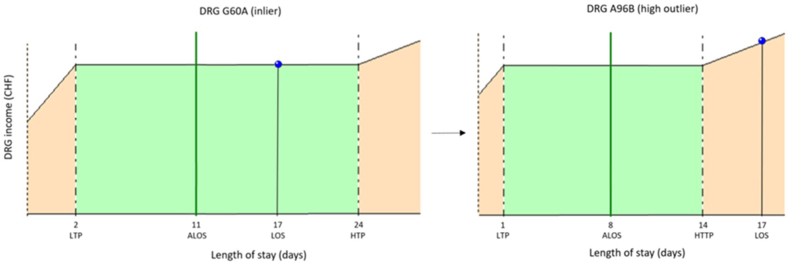
Examples of impact when adding CIM CHOP code 99.BC.12 From SwissDRG G60A inlier to SwissDRG A96B high outlier. The initial DRG G60A (Neoformation of digestive organs, >1 day of hospitalisation, with severe level of complication or comorbidity) with an inlier CW of 1.386 point (CHF 14 761) is pushed into DRG A96B when CHOP code 99.BC.12 is added to the list of codes, resulting in a high outlier CW of 1.379 (CHF 14 686). The high outlier CW of DRG A96B is obtained by adding 0.088 point of CW per day for each day since the 14th day (HTP) to the inlier CW (1.027 + (4x0.088) = 1.379). There is a compensation, and the loss of revenue is limited to CHF 74.

**Fig. 2c:**
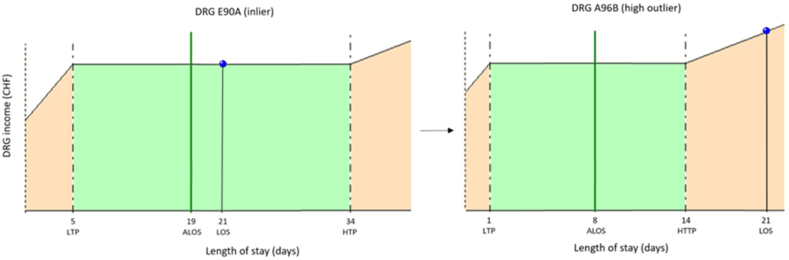
Examples of impact when adding CIM CHOP code 99.BC.12 From SwissDRG E90A to SwissDRG A96B high outlier The initial DRG E90A (Disease of respiratory organs with complex treatment in intensive care unit) with an inlier CW of 4.999 points (CHF 53 240) is pushed into DRG A96B when CHOP code 99.BC.12 is added to the list of codes, resulting in a high outlier CW of 1.731 point. The high outlier CW of DRG A96B is obtained by adding 0.088 point of CW per day for each day since the 14th day (HTP) to the inlier CW of DRG A96B (1.027 + (8x0.088) = 1.731 or CHF 18 435). The difference is a loss of revenue of CHF 34 804.ALOS: Average LOS; LTP: Lower Trim Point; HTP: Higher Trim Point; DRG: Diagnosis Related Group.

Among the 89 cases that changed DRGs with the addition of CIM CHOP code 99.BC.12, 42 DRG A96B generated a revenue loss of 83.149 points of CW (total loss of CHF 885 537), while 47 DRG A96B had a positive impact on the revenue of 11.165 points of CW (total gain of CHF 118 907), corresponding to a net loss of revenue of 71.984 points of CW or CHF 766 630.

Simulation with CIM CHOP code 99.BC.14.

Adding the CIM CHOP code 99.BC.14 (equal or more than 50 CIM) did not change the initial DRG and CW in 182 cases (66 %). In the remaining 93 cases (34 %), the initial SwissDRG was affected by the addition of the code. In 41/93 of these cases (44 %), the new CW was lower, while it was higher in the remaining 52 cases (56 %).

The addition of the CIM CHOP code 99.BC.14 generated a loss of revenue of 55.5 points of CW or a total loss of CHF 591 554. The largest single positive effect of adding a CIM CHOP code 99.BC.14 was +0.977 point of CW (mean: +0.518; range: +0.019 to +0.977), while the largest single negative impact was −8.078 points of CW (mean: −2.012; range: −8.078 to −0.047). When factored into the SwissDRG grouper, the CIM CHOP code 99.BC.14 consistently altered the original DRG to DRG A96A (inlier CW of 1.578 point).

The initial DRGs positively impacted by the CIM CHOP code 99.BC.14 had a mean (SD) CW of 0.983 (0.402) point. The initial DRGs negatively impacted had a mean (SD) CW of 4.099 (2.73) points. As for CIM CHOP code 99.BC.12, an increasing negative revenue gap resulted with increasing CW categories after the addition of a 99.BC.14 CHOP code, however less important compared to code 99.BC.12. Among the 93 cases changing the DRG through the addition of CIM CHOP code 99.BC.14, 41 DRG A96A generated a revenue loss of 82.512 points of CW (total loss of CHF 878 752), while 52 DRG A96A had a positive impact on the revenue of 26.967 points of CW (total gain of CHF 287 199), corresponding to a net loss of revenue of 55.545 points of CW or CHF 591 554.

## 4 Discussion

This simulation study evaluated the economic impact of adding a code for CIM services provided to complex medical patients in a Swiss tertiary care facility. The revenue simulation revealed a significant loss after the addition of such a code, which adds to an already highly negative result for this population. This situation can be summarized as “reduced remuneration for additional work” for service providers and is due to an unbalanced algorithm. The latter favors less complex patients (generating DRGs with lower CWs) over complex patients generating DRGs with high CWs. Invariably, CHOP code 99.BC.12 lead the SwissDRG grouper algorithm to select the DRG A96B, which had an inlier CW of 1.027 point in 2021. Therefore, for complex and severe cases, yielding a DRG with a high CW before coding CIM services, bigger revenue losses were observed. While the CHOP code 99.BC.11 had no impact, a negative effect was observed for both code 99.BC.14 and for code 99.BC.12, albeit with a smaller overall simulated loss of revenue. The case mix of polymorbid and complex patients within a tertiary referral centre may hence be particularly negatively affected. More broadly, sustainability of CIM for centres providing these innovative services to complex patients requires a rapid revision of the SwissDRG grouping algorithm.

The SwissDRG PPS intends to encourage efficient use of resources for inpatient care. The system can be considered as balanced if the average costs of inpatient stays in a specific DRG are offset by the premium. However, care providers may have higher costs not entirely covered by the planned reimbursement, leading to imbalance. This situation mostly occurs in tertiary academic medical centres, and is related to the vulnerable patient population, an expanded health care mandate or to the deployment of new treatment strategies [9]. These situations generate rare DRGs, outlier DRGs or high variations in costs, ultimately leading to an inadequate estimate of the DRG-related CW and therefore of the planned reimbursement [9,24].

In 2021, 1 439 968 cases were billed with SwissDRG PPS by hospitals in Switzerland, among which 614 were categorized under DRG A96A (0.04 %) and 451 to DRG A96B (0.03 %) [25]. Several studies have documented the widespread and increasing use of CIM in academic centres [19,26]. Lausanne University Hospital is one of the first Swiss tertiary academic hospital delivering structured specialized inpatient CIM care through a dedicated Centre for Integrative and Complementary Medicine. CIM has not only been effectively implemented in academic settings but has also shown usefulness for patients dealing with severe, recurring or chronic illnesses [16,17,27]. However, the present study demonstrates that the currently applied SwissDRG grouping algorithm underfunds and thus discourages these treatments: if the CIM CHOP code 99.BC.12 were used for the eligible population in 2021, the result would have been a loss of revenue of more than CHF 760 000 for the CHUV in a population with an already highly negative net balance for the hospital. CIM CHOP code 99.BC.11 would have had no impact and CIM CHOP code 99.BC.14 would also have generated a loss of revenue of more than CHF 590 000. This result demonstrates that CIM services are not properly valued in tertiary facilities mostly dealing with complex cases assigned to DRGs with high CWs.

The patient base for CIM therapies has evolved over time, becoming more medically complex and diverse in academic settings, yet the Swiss PPS's grouper has not kept pace with this evolution. This is extremely unfortunate for the patients, representing missed opportunities for cost savings [[12], [13], [14], [15]]. Moreover, there is an incentive to offer CIM services only to cases with advantageous low CW DRGs. Indeed, only two DRGs dedicated to CIM therapies with a CW near to 1 are available (A96A with an inlier CW = 1.578 and A96B with an inlier CW = 1.027). Pushing the initial CW, driven by the patient's diagnoses and obtained without a CIM CHOP code 99.BC, into DRG A96A or A96B after adding a CIM CHOP code leads to the following unintended effect: the higher the initial CW compared to the CW of the CIM dedicated DRGs, the higher the loss of income (Fig. 1). This de facto prejudices seriously ill patients who may benefit from integrative medicine.

Under normal circumstances, CW is a representation of the costs incurred by providers for treating a particular case and therefore the complexity of the case. Thus, moving from a CW of 4 or higher to a CW near 1 involves reducing the cost evaluation by a factor of 4 or more. This tends to render CIM services financially inaccessible for many of the severely ill and complex cases commonly encountered in academic settings.

Like most countries in the world, Switzerland finances outliers based on a per day additional charge once the upper limit of LOS (high trim point) is exceeded for the DRG considered [5,7]. However, for DRGs with high CWs, the adjustment is by far not sufficient to compensate for the drop in cost weight due to the DRG change induced by CIM CHOP code 99.BC.12 (Fig. 2a, Fig. 2b, Fig. 2cA, 2B and 2C).

Differences between revenues and costs are integral part of PPSs. Ideally, profitable cases compensate for cases generating a deficit for the hospital. However, in reality, cases are distributed asymmetrically among hospitals, which does not allow this mechanism to operate fairly [9,28,29].

The reduction in revenue loss with code 99.BC.14 (≥50 CIM sessions) compared to code 99.BC.12 (10–25 CIM sessions) implies that the algorithm is working as it should, allowing for the additional resources involved in more treatment sessions. Cases are thus classified in DRG A96A (CW inlier of 1.578) with a code 99.BC.14, whereas they are classified in DRG A96B (CW inlier of 1.027) with a code 99.BC.12. The problem lies upstream, when the initial DRG is moved to DRG A96A or A96B by a classifying CIM CHOP code, without considering the initial CW of the case.

The proposal at this point would be to exclude all DRGs that have a cost weight greater than the DRG A96A or A96B from potential grouping in one of these two CIM DRGs. This solution would not impact the present fair reimbursement of cases meriting a CW from the CIM-specific DRGs, while also preventing significant reductions in the CW for expensive cases. Providers willing to invest resources in the delivery and justification of CIM therapies will be able to do so at the maximum cost of their decided investment and no more. The key investment is to create and operate a multidisciplinary care team, according to the CHOP code requirements [30]. This team must be led by a certified CIM physician and should consist of several therapists and specialized nurses trained in the field of CIM. Ultimately, the expenses reported by these providers will contribute to shaping an equitable reimbursement strategy moving forward.

## 5 Limitations

For the purpose of the simulation and since we do not dispose of a specific document justifying the resources used, we considered every patient treated by the CEMIC. Therefore, all patients who received CIM therapies in 2021 by the CEMIC at Lausanne University Hospital were included in the simulation, regardless of the number of sessions these patients had. This may exaggerate the increase in financial losses for this particular population, but it does not change the conclusion drawn about the DRG grouper's algorithm. Indeed, a case going from a CW of 7 to a CW four times lower is already one case too many.

## 6 Conclusions

A PPS creates motivations for hospitals to manage patient treatment cost-efficiently. It is crucial to avoid adverse financial incentives that could jeopardize both patient care and the advancement of new therapeutic approaches aimed at potential savings. New value-added and innovative care protocols such as CIM pathways need a reactive strategy from the PPS to adjust the fee. Simulations should be better integrated alongside to standard CW adjustment based on previous billing period to shorten the reaction time and limit the losses for the providers.

## Ethics and other permissions

Not applicable. No patient health-related information is discussed neither gathered in a database for the present study. This study is a financial simulation which analyses the reimbursement of complementary medicine in Switzerland. The sole parameters utilized in this research are the diagnostic related group (DRG) and its associated cost weight (CW) based on the 2021 definitions of the German modified International Classification od Disease (ICD-10-GM), along with hospital reimbursement (revenue) in Swiss Francs (CHF) and expenses (cost) related the hospital stay. These data were collected through inquiries to the hospital's electronic coding system (Medstat) without accessing the medical health record of patients. We therefore used an anonymized database containing DRG, CW, revenue and costs in CHF of patients who have had a CEMIC treatment.

## Funding

No funding sources.

## Data availability

The datasets used and/or analysed during the current study have not been deposited into a publicly available repository. Data will be made available on request.

## CRediT authorship contribution statement

**Fabian Grass:** Writing – review & editing, Writing – original draft, Methodology, Formal analysis, Data curation. **Chantal Berna:** Writing – review & editing, Writing – original draft, Methodology, Formal analysis, Data curation. **Charles-André Vogel:** Writing – review & editing, Writing – original draft, Validation, Methodology. **Nicolas Demartines:** Writing – review & editing, Writing – original draft, Supervision, Methodology. **Fabio Agri:** Writing – review & editing, Writing – original draft, Methodology, Formal analysis, Data curation, Conceptualization.

## Declaration of competing interest

The authors declare that they have no known competing financial interests or personal relationships that could have appeared to influence the work reported in this paper.
